# Pharmacological evaluation of levofloxacin residue depletion and hemato-biochemical alterations in broiler chickens using a validated HPLC method

**DOI:** 10.1038/s41598-025-17575-0

**Published:** 2025-09-12

**Authors:** Ahmed E. A. Mostafa

**Affiliations:** Department of Preclinical Veterinary Medical Sciences (Pharmacology), Faculty of Veterinary Medicine, Delta University for Science and Technology, Gamasa City, Dakahlia Governorate Egypt

**Keywords:** Levofloxacin residues, Broiler chickens, Withdrawal period, Hematological and biochemical effects, High-performance liquid chromatography (HPLC), Food safety, Drug discovery, Physiology, Diseases, Medical research

## Abstract

The extensive use of antibiotics in poultry farming has raised significant concerns regarding drug residues in edible tissues and their implications for public health. This study investigates the withdrawal period and physiological effects of levofloxacin following intramuscular administration in broiler chickens. Forty healthy male broilers were divided into four groups to receive levofloxacin at 10 mg/kg body weight for four consecutive days, with sampling performed at days 5, 7, and 9 post-treatment. Residues of levofloxacin in liver, kidney, and breast muscle were quantified using high-performance liquid chromatography (HPLC). Hematological and biochemical parameters were also evaluated, including liver and kidney function markers and antioxidant enzymes. The results revealed a significant presence of levofloxacin residues in all tissues on days 5 and 7, with levels falling below maximum residue limits by day 9. Hematological analysis showed temporary alterations in red and white blood cell counts, while biochemical tests indicated transient elevations in liver enzymes and oxidative stress markers, which normalized by day 9. These findings highlight the importance of observing an adequate withdrawal period to ensure food safety and support the recommendation that broiler tissues are safe for human consumption nine days after the last dose of levofloxacin. All animal procedures were approved by the Research Ethics Committee of the Faculty of Pharmacy, Delta University (Approval No. FPDU14/2024) and followed the ARRIVE guidelines. The findings provide practical guidance for poultry farmers to ensure compliance with food safety regulations and withdrawal periods.

**Supplementary Information:**

The online version contains supplementary material available at 10.1038/s41598-025-17575-0.

## Introduction

The treatment of broilers with a wide range of veterinary antibiotics often results in detectable residues in edible tissues and eggs, these residues have direct effect on customers by embracing some conjugates and/or metabolites. Using more antibiotics might cause indirect effect through resistance to some strains of microorganisms^1^.

All poultry are medicated by different types of antibiotics and the residues detected in meat, and that must be not exceed the recommended residues limits^2^.

Fluoroquinolones consists of wide range of antimicrobial agents which used in human and animal’s medication. It’s action through inhibiting the activity of ribonucleic acid gyrase enzyme and so, preventing the supercoiling process of bacterial DNA. As a result, they have a broad spectrum activity against gram-negative and gram-positive microorganism, pseudomonas spp. and Rickettsia, and with a great action against beta-lactamase producing microorganisms^3^.

There are different generations of fluoroquinolones from these, the levofloxacin which considered the third generation of fluoroquinolone which associate with the optical compound of ofloxacin That consist of two-fold higher activity that the parent compound, Currently it used with wide range in different human diseases including higher and lower respiratory tract, urogenital apparatus, skin and soft tissue and used in food producing animals^4^. Like all quinlones, levofloxacin act as a broad-spectrum antibiotic that act against gram-positive and gram-negative microorganisms. It’s action by obstruction the activity of ribonucleic acid gyrase enzyme and topoisomerase IV, 2 microorganism type II topoisomerases^5^. Levofloxacin apply it’s bactericidal effect by stopping bacterial DNA from duplicating by inhibiting the ligase action of the type II topoisomerases, gyrase, and topoisomerase IV, which prevent DNA to supercoiling and with their ligase activity interrupted. And so, DNA with single- and double-strand breaks released that lead to bacterial death^6^. With the wide usage of fluroquinolones without conceren on the withdrawal time from the different tissue might accumulation of residues in food used for human consumption. These residues considered a great risk to public health by formation microorganism resistance ^1^.Detection of antibacterials residues in food with high specificity and sensitivity by using high performance liquid chromatography which is widely used to detect the numerous quantity of antibiotic residues^7^.

Recent advances in analytical methods have significantly improved the detection and quantification of quinolone residues in animal-derived food products. Studies such as those by Li et al.^8^^,^ Lin et al.^9^^,^ and Hu et al.^10^ have introduced enhanced sample preparation and detection strategies, including magnetic solid-phase extraction and pH-dependent liquid–liquid extraction, which offer higher sensitivity and specificity in detecting fluoroquinolone residues. These developments underscore the importance of using validated and robust analytical techniques, such as HPLC, to monitor antibiotic residues effectively.

Levofloxacin was selected for this study due to its favorable pharmacokinetic profile compared to other fluoroquinolones. It exhibits higher oral bioavailability, prolonged half-life, enhanced tissue penetration (particularly in lung and muscle tissues), and a broader antimicrobial spectrum. These advantages make levofloxacin a frequently used therapeutic option in poultry production^11^^,^^12^. Alternative strategies have also been explored to reduce the incidence of necrotic enteritis caused by *Clostridium perfringens*, including the use of probiotics^13^.

Despite the widespread use of levofloxacin in poultry, few studies have simultaneously investigated its residue depletion along with hematological and biochemical alterations in broiler chickens using validated HPLC methods. Therefore, this study addresses this gap by integrating pharmacokinetic and physiological assessments.

Aim of the Work:

This study aimed to:

1. Determine the withdrawal time of levofloxacin from liver, kidneys, and breast muscles of broilers after intramuscular injection (I/M) of the drug (10 mg/kg for four consecutive days) by using HPLC.
2. Evaluate some hematological parameters (CBC) after intramuscular injection (I/M) of the drug (10 mg/kg throughout the treatment period).
3. Evaluate some biochemical parameters as liver function enzymes (AST, ALT), kidney function enzymes (blood serum urea, creatinine) and antioxidants enzymes (SOD, GPX, CAT).

## Materials and methods

### Drugs

**Fig. 1:**
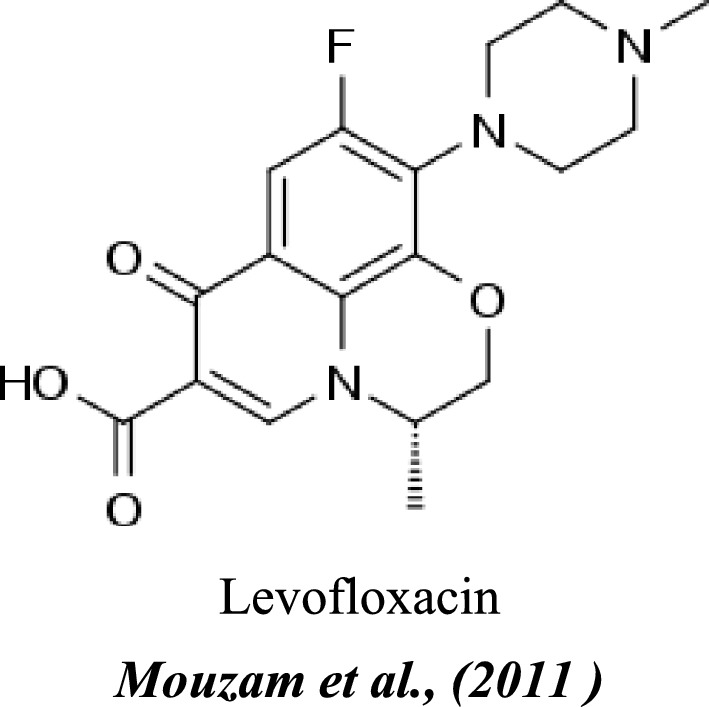
Structural formula of levofloxacin.

- Product Name and Source:
  - Levoxin^®^ (Levofloxacin hemihydrate 0.5%) was obtained as a 100 mL vial (5 mg/mL) from Amoun Pharmaceutical Ltd., Egypt.
- Pharmacological Classification:
  - Levofloxacin is a broad-spectrum third-generation fluoroquinolone antibiotic and the levo-isomer of ofloxacin^14^^,^^15^.
- Dosage Rationale:
  - A dose of 10 mg/kg body weight for four consecutive days was selected, reflecting a common regimen in poultry farms for treating respiratory and systemic infections, and has been previously validated^12^.
- Chemical and Physical Properties:
  o Chemical Name: (3S)-9-Fluoro-3-methyl-10-(4-methyl-1-piperazinyl)-7-oxo-2,3-dihydro-7H-[1,4]oxazino[2,3,4-ij]quinoline-6-carboxylic acid
  o Empirical Formula: C₁₈H₂₀FN₃O₄
  o Molecular Weight: 361.37 g/mol
  o Appearance: Dull-yellow crystalline antibiotic powder
  o Solubility: Soluble in cold water
  o Formulation: Sterile solution containing 5 mg/mL levofloxacin base^11^^,^^16^
- The structural formula of levofloxacin is shown in Fig. 1.

### Experimental animals and housing conditions

The study was conducted in (n = 40) healthy Rock-3(IR-3)—on 20-day-old chickens , a strain of broiler chicken 4 groups at a weight 1 kg ± 50 gm weight developed in Elabrar poultry company, Abohamad, Egypt. The birds were kept under similar conditions and were housed in post graduate research laboratory in Faculty of Veterinary Medicine, Zagazig University and were humanely handled.

Only male broiler chickens were used in this study to minimize the variability associated with hormonal fluctuations and sex-based metabolic differences that could potentially influence drug pharmacokinetics and tissue residue depletion^17^.

The broilers were kept underneath observation for one week before commencement of experiment at the faculty experimental farm (faculty of vet. Medicine zagazig university) and subjected to clinical examination so as to exclude the likelihood of illness. The birds were provided antibiotic-free normal broiler feed for fourteen days. The poultry house was maintained at temperature farm house temperature. Feed and water were provided ad libtum and normal managemental practices were followed to stay the birds free from stress.The experiment was performed in accordance with the rules set by the moral Committee of Zagazig University, Egypt.

### Experimental design

Fourty healthy male broiler chickens were randomly allocated to four groups (n = 10 per group). Analyses were performed blinded to treatment group where feasible. The birds were assigned to four treatment groups as follows:

- Group 1 (Control): Untreated control group.
- Group 2: Received intramuscular (IM) injections of levofloxacin (Levoxin® vial) at a dose of 10 mg/kg body weight for four consecutive days; slaughtered on Day 5 post-injection.
- Group 3: Received the same levofloxacin treatment; slaughtered on Day 7 post-injection.
- Group 4: Received the same levofloxacin treatment; slaughtered on Day 9 post-injection.

### Sampling procedures

At the time of slaughter, tissue samples were collected from each bird:

- Breast muscle (100 g)
- Liver (50 g)
- Kidney (5 g)

Samples were stored at − 20 °C until analysis by High-Performance Liquid Chromatography (HPLC).

Sampling was conducted on Days 5, 7, and 9 post-treatment to monitor the depletion phase of levofloxacin. Early time points (e.g., Day 1 or 3) were not included to minimize animal sacrifice during peak drug accumulation. However, future studies should consider additional early and late time points to establish a more comprehensive depletion curve^18^.

A. Hematological Analysis:

Blood was collected in heparinized tubes (2 mL) for hematological evaluation.

B. Biochemical Analysis:

Whole blood was also collected in plain tubes (3 mL) for serum separation. Samples were centrifuged at 3000 rpm for 10 min within one hour of collection. Serum was transferred to sterile vials and stored appropriately.

### Hematological and biochemical analysis

Hematological and Biochemical Analysis

Blood samples were drawn from the jugular vein at Days 5, 7, and 9 post-treatment. Parameters assessed included:

- Hemoglobin (Hb): Sahli’s acid hematin method
- Packed Cell Volume (PCV)
- Total Leukocyte Count (TLC)
- Differential Leukocyte Count (DLC)

Hematological analysis was conducted using the Rayto 7200 Auto Hematology Analyzer (MEDINICS^®^).

1. Liver Function Tests:

- Aspartate aminotransferase (AST)
- Alanine aminotransferase (ALT)
- *Measured using Chem 7 Semi-Automated Clinical Chemistry Analyzer (Erba®)*

2. Kidney Function Tests:

- Serum urea
- Serum creatinine
- *Analyzed using Chem 7 Analyzer (Erba* ^*®*^ *)*

3. Antioxidant Enzymes:

- Catalase (CAT)
- Glutathione Peroxidase (GPx)
- Superoxide Dismutase (SOD)
- *Measured using RT-2100C Microplate Reader (Rayto* ^*®*^ *)*

### High-performance liquid chromatography (HPLC)

#### Sample preparation for residue analysis

1. Weigh 3 g tissue
2. Add 15 mL extraction solution
3. Shake 1 min
4. Centrifuge (15,000 rpm, 8 min)
5. Hydrolyze (50 °C, 90 min)
6. Cool and centrifuge (5000 rpm, 10 min)
7. Inject 50 µL into HPLC

#### Preparation of standards

Chromatographic separation was performed on a Hypersil ODS C18 column (150 × 4.6 mm, 5 µm) at 50°C. Mobile phase: acetonitrile:water (12:88, v/v) containing 0.75% formic acid and 0.4% triethylamine. Flow rate 1.0 mL/min; injection volume 50 µL; detection at 295 nm; run time 8 min; retention time for levofloxacin ≈ 3.341 min. as following:

- Stock: 1 mg/mL
- Working: 0.025–1 µg/g
- Flow rate: 1 mL/min
- Column temp: 50 °C
- Detection: 295 nm
- Mobile phase: 12% acetonitrile, 0.75% formic acid, 0.4% triethylamine in water

### HPLC method validation

Method validation was performed according to ICH Q2(R1) guidelines. Linearity, system precision, method precision, accuracy (recovery %), selectivity, LOD/LOQ, ruggedness and robustness were evaluated as described; acceptance criteria and obtained values are reported in Section "HPLC method validation" (as per ICH Guidelines):

- System Precision: 5 replicates, RSD ≤ 1%
- Linearity and Range: r2 ≥ 0.99
- Method Precision: 5 replicates, RSD ≤ 1%
- Selectivity: no interference in control tissue
- Accuracy and Recovery: spiking and comparison with known standards
- LOD and LOQ: 3:1 and 10:1 signal/noise ratios
- Ruggedness & Robustness: RSD ≤ 6% under varying conditions

LOD and LOQ were determined using signal-to-noise ratios of 3 and 10, respectively. The LOD and LOQ were 0.003 µg/g and 0.01 µg/g, respectively.

Detailed HPLC equipment, setup and chemical reagents and solvents were provided in the Supplementary Material (Section 1).

General lab instruments used for processing and analysis are listed in the Supplementary Material (Section 2).

### Euthanasia protocol

Chicks were humanely euthanized using inhalation of isoflurane (5% concentration in oxygen) administered via a closed chamber until complete loss of consciousness. This was followed by cervical dislocation as a secondary physical method to ensure death. The procedure was performed by a trained professional. This method was approved by the Research Ethics Committee at the Faculty of Pharmacy, Delta University for Science and Technology (Approval No. FPDU14/2024), and complies with both institutional guidelines and the ARRIVE guidelines for the humane treatment of animals.

### Statistical analysis

Data were analyzed using one-way Analysis of Variance (ANOVA) followed by Tukey’s post hoc test for multiple comparisons to assess significant differences among groups. Statistical significance was considered at p < 0.05. All data were expressed as mean ± standard Error (SE).

$${\text{Standard}}\;{\text{error}}\;(\overline{X} ) = \sqrt {\frac{{S_{X}^{2} + \overline{X}^{2} }}{n}}$$

Statistical analyses were performed using GraphPad Prism 9 (GraphPad Software, USA). Normality was tested with Shapiro–Wilk; homogeneity with Levene’s test. One-way ANOVA followed by Tukey’s post hoc test was used for multiple comparisons; non-normal data were log-transformed or analysed with non-parametric tests where applicable. Results are presented as mean ± SE; p < 0.05 considered significant.

### Ethical approval and ARRIVE guidelines

All animal experimental procedures were approved by the Research Ethics Committee at the Faculty of Pharmacy, Delta University for Science and Technology (FPDU-REC), under approval number FPDU25/2025. The ethical approval was granted specifically for the animal study component of the research protocol.

All methods involving animals are reported in accordance with the ARRIVE guidelines (https://arriveguidelines.org) to ensure transparent and comprehensive reporting of animal research.

## Results

### Effect of levofloxacin injection on hematological parameters in broilers

A) Effect on Total Erythrocytic Count

**Fig. 2:**
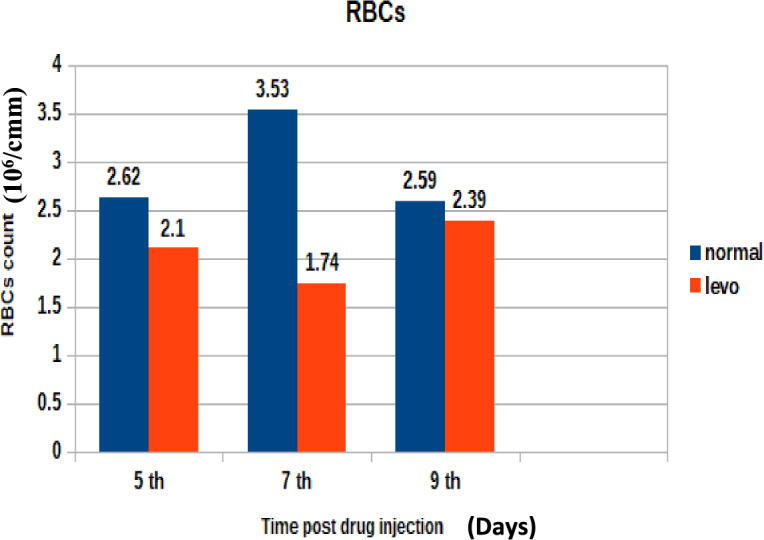
The effect of levofloxacin (10 mg/kg.b.wt) I/M injection throughout the treatment course on total erythrocytic count (10^6^/cmm) in broilers at 5th, 7th and 9th day post last injection.

The changes in total erythrocyte count across the study groups are presented in Fig. 2. It was found that broilers injected with levofloxacin throughout the treatment course showed significant (p < 0.05) decrease in total erythrocytic count at 5th and 7th post last day of injection besides insignificant decrease in total erythrocytic count at 9th day post last day of injection when compared with control broilers.

B) Effect on Total leukocytic Count

**Fig. 3:**
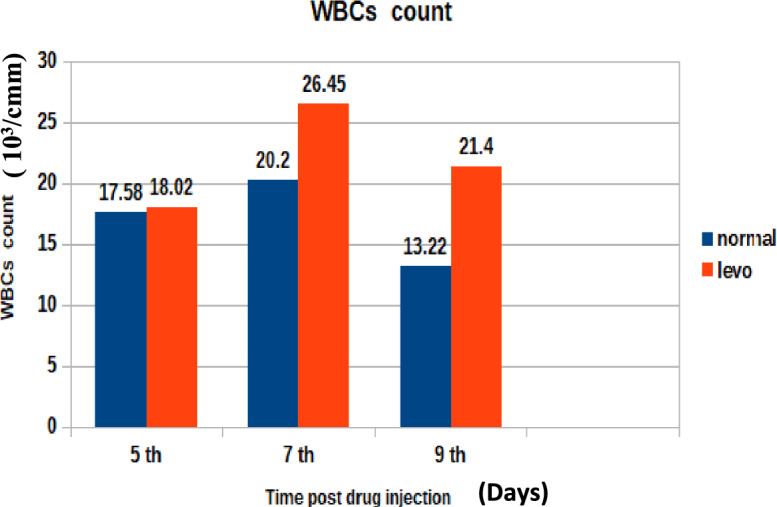
The effect of levofloxacin (10 mg/kg.b.wt) (I/M) injection throughout the treatment course on total leukocytic count (× 10^3^/cmm) in chicks at 5th, 7th and 9th day post last injection.

The elevation in leukocyte count following treatment is illustrated in Fig. 3. It was found that broilers injected with levofloxacin (10 mg/kg.b.wt) throughout the treatment period showed significant (p < 0.05) increase in total leukocytic count at 5th and 7th post last day of injection besides insignificant increase in total leukocytic count at 5th day post injection when compared with control broilers.

C) Effect on Total hemoglobin content

**Fig. 4:**
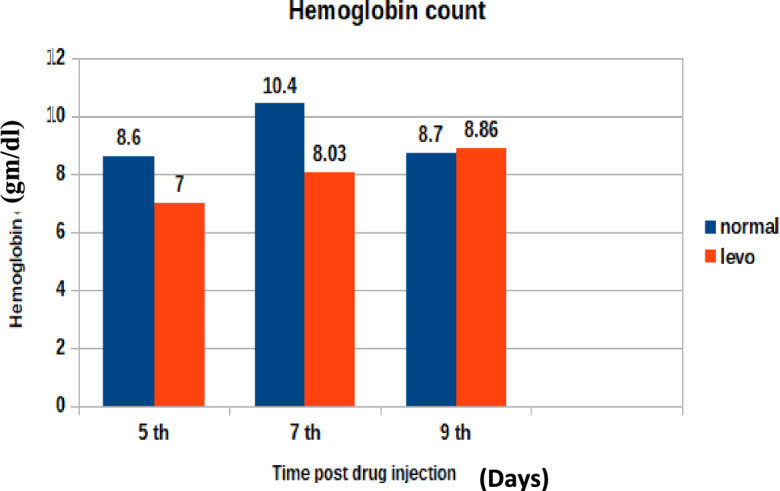
The effect of levofloxacin (10 mg/kg.b.wt) (I/M) injection throughout the treatment course on hemoglobin count (gm/dl) in chicks at 5th, 7th and 9th day post last injection.

Hemoglobin fluctuations across the study days are shown in Fig. 4. It was found that broilers injected with levofloxacin (10 mg/kg.b.wt) throughout the treatment course showed significant (p < 0.05) decrease in hemoglobin content at 5th and 7th post last day of injection besides insignificant increase in hemoglobin content at the 9th day post last day of injection when compared with control broilers.

#### Effect on Some liver function tests:

Liver enzymes (AST, ALT, and ALP)

1. Aspartate aminotransferase (U/L)

**Fig. 5:**
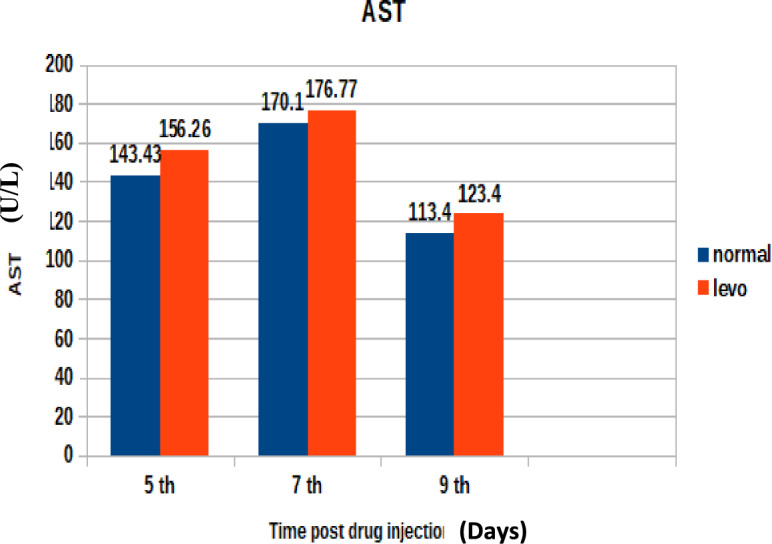
The effect of levofloxacin (10 mg/kg.b.wt) (I/M) injection throughout the treatment course on Aspartate Aminotransferase (U/L) in broilers at 5th, 7th and 9th day post last injection.

Figure 5 demonstrates the AST activity over the experimental period. It was found that broilers injected with levofloxacin (10 mg/kg.b.wt) throughout the treatment course showed significant (p < 0.05) increase in Aspartate aminotransferase (U/L) at 5th and 7th post last day injection besides insignificant Increase in Aspartate aminotransferase (U/L) at 9^th^ day post injection when compared with control broilers .

2- Alanine aminotransferase (U/L)

**Fig. 6:**
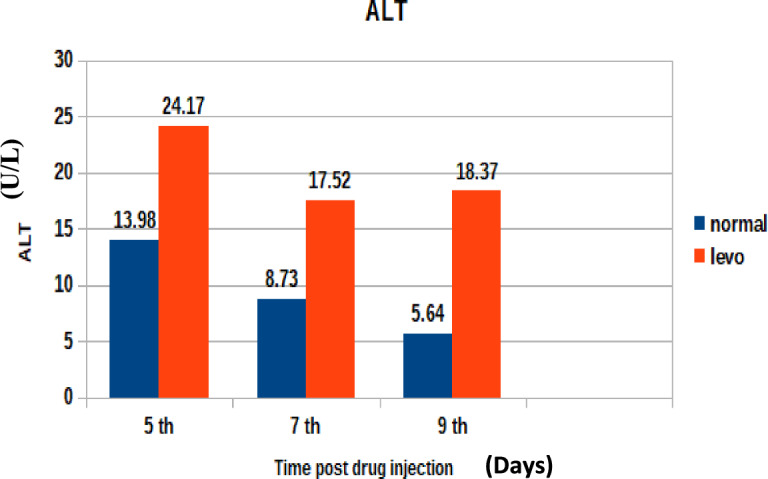
The effect of levofloxacin (10 mg/kg.b.wt) I/M injection for 4 consecutive days on Alanine aminotransferase (U/L) in broilers at 5th, 7th and 9th day post last injection.

Figure 6 illustrates the levels of ALT in treated and control broilers. It was found that broilers injected with levofloxacin throughout the treatment course showed significant (p < 0.05) increase in Alanine aminotransferase (U/L) at 5th and 7th post last day injection besides insignificant increase in Alanine aminotransferase (U/L) at 9th day post last day of injection when compared control broilers.

3- Alkaline phosphatase (U/L)

**Fig. 7:**
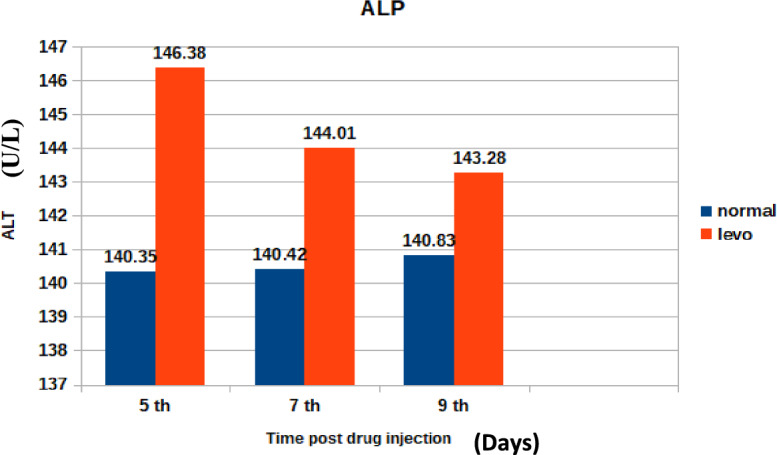
The effect of levofloxacin (10 mg/kg.b.wt) (I/M) injection throughout the treatment course on Alkaline phosphatase (U/L) in broilers at 5th, 7th and 9th day post last injection.

Alkaline phosphatase activity is presented in Fig. 7. It was found that broilers injected with levofloxacin (10 mg/kg.b.wt) throughout the treatment course showed significant (p < 0.05) increase in Alkaline phosphatase (U/L)at 5^th^ and 7^th^ post last day injection besides insignificant increase in Alkaline phosphatase (U/L) at 9^th^ day post injection when compared with control broilers .

Effect on Some kidney function tests:

1. Serum urea (mg/dl)

**Fig. 8:**
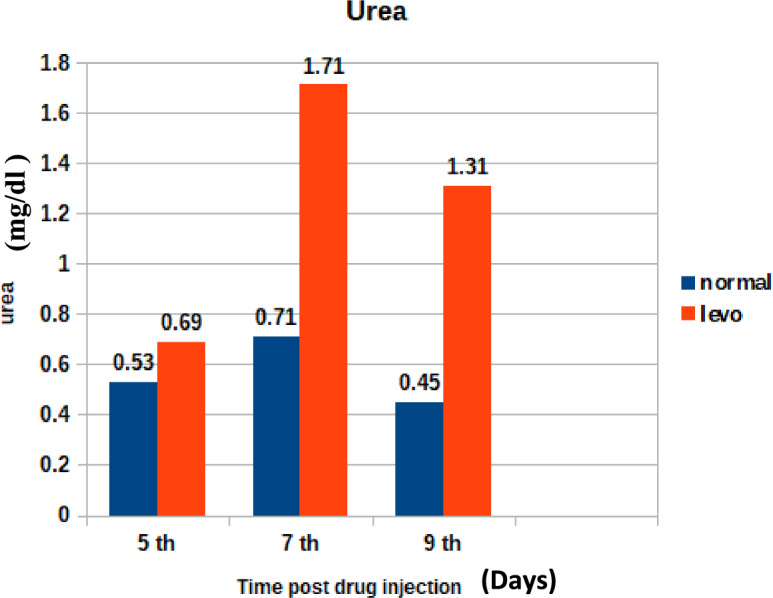
The effect of levofloxacin (10 mg/kg.b.wt) (I/M) injection throughout the treatment course on serum urea (mg/dl)in broilers at 5th, 7th and 9th day post last injection.

Figure 8 shows the changes in serum urea levels post-treatment. It was found that chicks injected with levofloxacin (10 mg/kg.b.wt) throughout the treatment course showed significant (p < 0.05) Increase in Serum urea (mg/dl)at 5th and 7th post last day injection besides insignificant increase in Serum urea (mg/dl) at 9th day post last day injection when compared with control broilers.

2. Serum Creatinine (mg/dl)

**Fig. 9:**
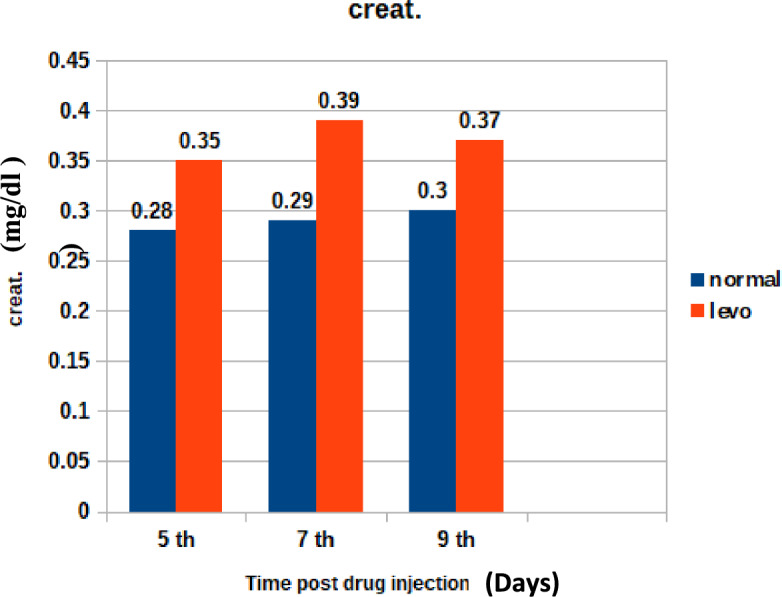
The effect of levofloxacin (10 mg/kg.b.wt) (I/M) injection for 5 consecutive days on Serum Creatinine (mg/dl) in broilers at 5th, 7th and 9th day post last injection.

Figure 9 highlights creatinine concentration trends over the trial. It was found that chicks injected with levofloxacin throughout the treatment course showed significant (p < 0.05) increase in Serum Creatinine (mg/dl)at 5th and 7th post last day injection besides insignificant Increase in Serum Creatinine (mg/dl) at 9th day post last day injection when compared with control broilers.

Effect on enzymatic antioxidants:

1. Superoxide dismutase (SOD)

**Fig. 10:**
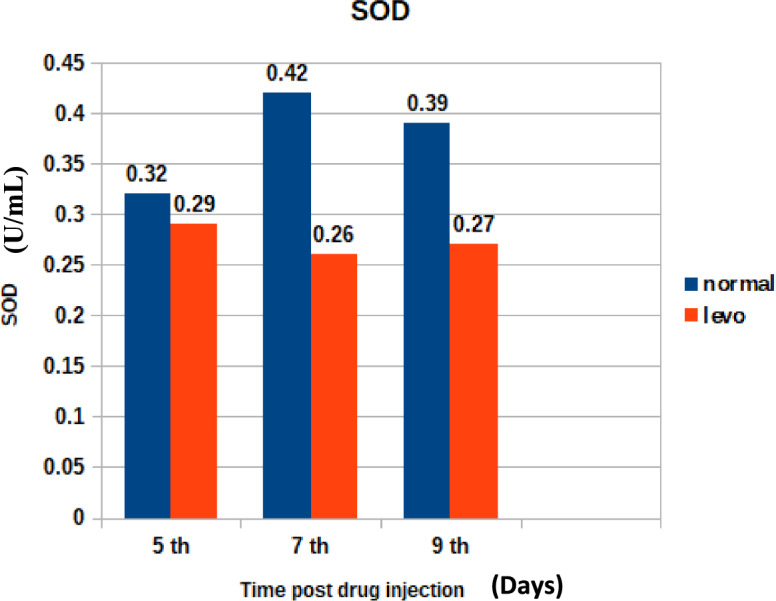
The effect of levofloxacin (10 mg/kg.b.wt) I/M injection throughout the treatment course on Superoxide dismutase (U/mL) in broilers at 5th, 7th and 9th day post last injection.

The effect on SOD levels is visualized in Fig. 10. It was found that chicks injected with levofloxacin (10 mg/kg.b.wt) throughout the treatment course showed significant (p < 0.05) decrease in Serum Superoxide dismutase (U/mL) at 5th and 7th post last day injection besides insignificant decrease in Superoxide dismutase (U/mL) at 9th day post last day injection when compared with control broilers.

2) Catalase (CAT)

**Fig. 11:**
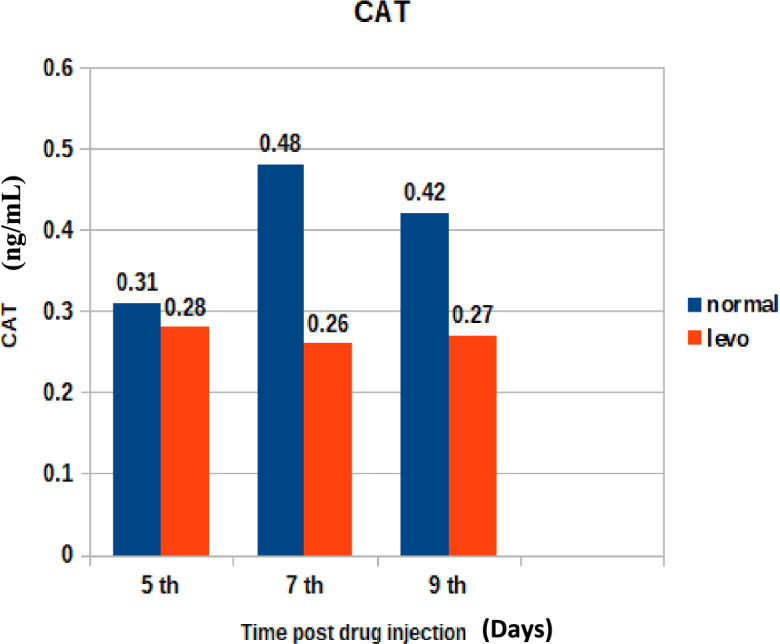
The effect of levofloxacin (10 mg/kg.b.wt) (I/M) injection throughout the treatment course on Catalase (ng/mL) in broilersat 5th, 7th and 9th day post last injection.

Figure 11 shows the serum catalase activity in broilers. It was found that chicks injected with levofloxacin (10 mg/kg.b.wt) throughout the treatment course showed significant (p < 0.05) decrease in Serum Catalase (ng/mL) at 5th and 7th post last day injection besides insignificant decrease in Catalase (ng/mL) at 9th day post last day injection when compared with control broilers.

3) Glutathione Peroxidase (GPX)

**Fig. 12:**
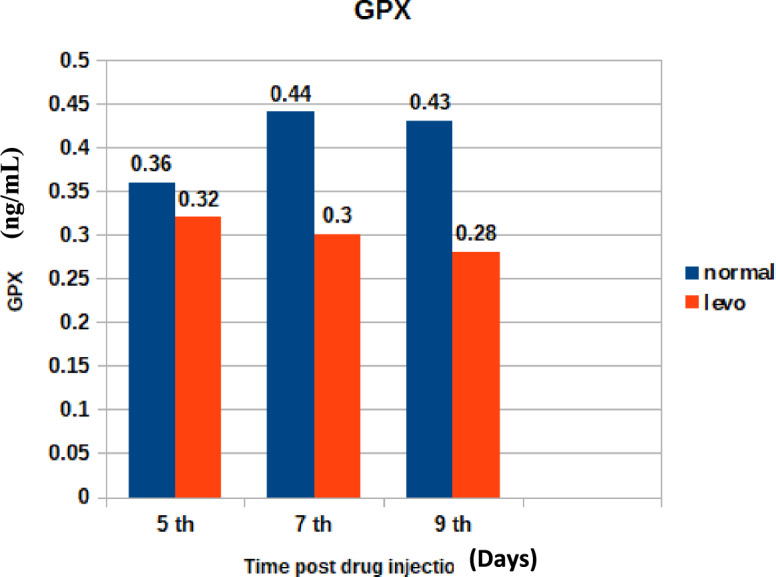
The effect of levofloxacin (10 mg/kg.b.wt)) (I/M) injection throughout the treatment course on Glutathione Peroxidase (GPX) (ng/mL) in chicks at 5^th^, 7^th^and 9^th^ day post last injection.

The depletion of GPX enzyme is illustrated in Fig. 12. It was found that chicks injected with levofloxacin throughout the treatment course showed significant (p < 0.05) decrease in Serum Glutathione Peroxidase (GPX) (ng/mL) at 5th and 7th post last day injection besides insignificant decrease in Glutathione Peroxidase (GPX) (ng/mL) at 9th day post last day injection when compared with control broilers.

### Levofloxacin residues

External standards were used for quantification during HPLC analysis. The method was validated for linearity, precision, accuracy, LOD, and LOQ in accordance with international guidelines to ensure reliability and reproducibility ^18^.

1. Method validation results:
2. System Precision:

The HPLC system is precise as the Relative Standard Deviation (RSD) of 5 replicates of toluene standard solution is ≤ 1% according to International conference on harmonization of technical requirements for registration of pharmaceuticals for human use (ICH).

1.2 Linearity and range:

Linearity of levofloxacin r^2^ = 0.99858.

1.3 Method Precision:

The method for levofloxacin separation is precise as the Relative Standard Deviation (RSD) of 6 replicates of marbofloxacin standard solution was 0.13%.

1.4 Selectivity and specificity:

There is no interference between the pure standard and peaks of any impurities or extracted solvents.

The retention time (R.T.) of levofloxacin was 3.341 min.

1.5 Accuracy and recovery:

The percentage recovery of levofloxacin spiked samples ranged from 98 to 101%.

1.6 Limit of detection (LOD):

LOD of levofloxacin was 0.003 µg µg/ml.

1.7 Limit of quantification (LOQ):

LOQ for levofloxacin was0.01 µg/ml.

1.8 Ruggedness:

The Pooled Relative Standard Deviation for levofloxacin was 2.8%.

1.9 Robustness:

The Pooled Relative Standard Deviation for levofloxacin was 2.1%.

**Fig. 13:**
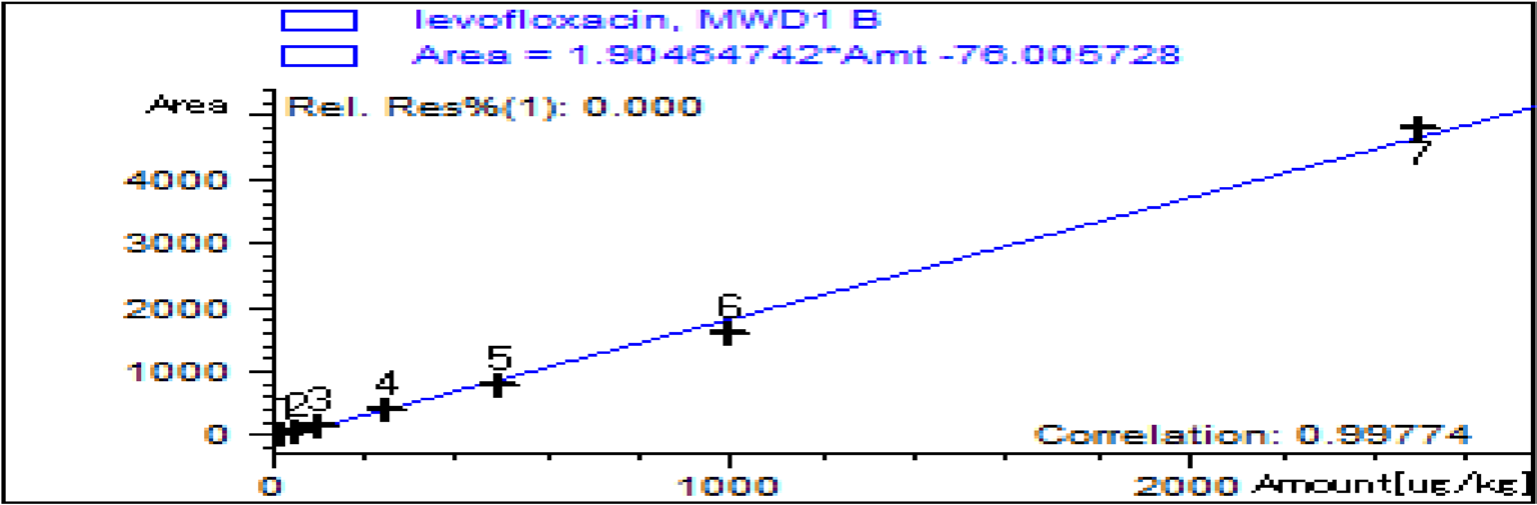
Standard curve of levofloxacin.

2. Standard curve preparation:

- The calibration curve for levofloxacin detection is shown in Fig. 13 . The concentrations of levofloxacin spiked tissues (µg/kg) and their corresponding peak responses are presented in Table 1.
- Calibration curve equation (area vs. concentration): y = 1905.96·x − 74.08; correlation coefficient r^2^ = 0.9949 (linearity 0.025–1.00 µg/g).
- Levofloxacin standard concentrations of 25, 50, 100,200,500 ,1000 and 2500 µg/gm were prepared in homogenized muscles of control chicks (blank samples) then treated according to the described extraction procedure and their corresponding peak responses (area under peak).
- The calibration curve was calculated by linear regression equation method as y = 76.005728 and x = 1.9048742 where y symbol indicated area under peak and x symbol indicated concentrations of levofloxacin.
- Linearity existed within range of 25 and 1 µg/kg with a correlation coefficient (r2 = 0.997).

**Table 1:** The concentrations of levofloxacin spiked tissues (µg/kg) and their corresponding peak response automatically using HPLC.

RT*	Level	Amount (µg/kg)	Area
3.341	1	25	31.50
	2	50	63.78
	3	100	136.26
	4	200	413.69
	5	500	782.59
	6	1000	1600.2
	7	25,000	4792.0

Values are mean ± SE (n = 10). (p < 0.05, one-way ANOVA followed by Tukey’s test). Units: µg/kg.

**Fig. 14:**
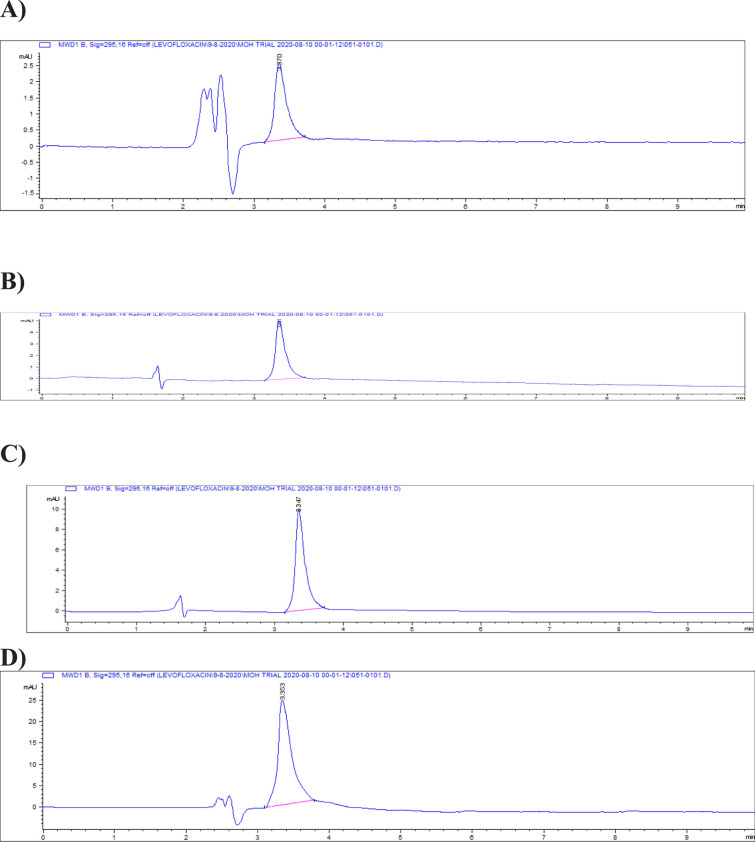

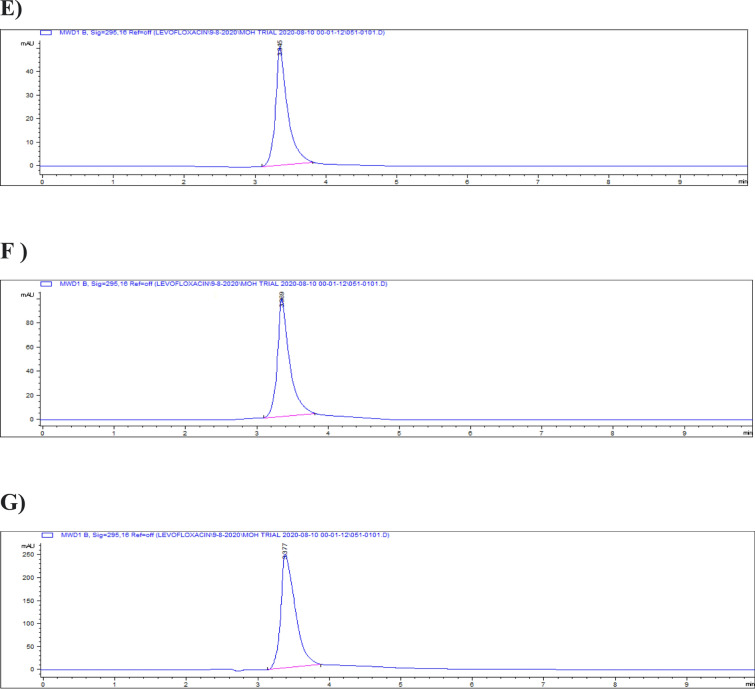
Chromatograms of levofloxacin spiked samples **a** 0.025, **b** 0.05, **c** 0.1, **d** 0.2, **e** 0.5 and **f** 1 and **g** 2.5 µg/gm.

Representative chromatograms for levofloxacin spiked samples are shown in Fig. 14.

On Day 9, none of the tissue samples exceeded the maximum residue limits (MRLs) established by regulatory authorities. All birds demonstrated residue levels well below safety thresholds.

## Discussion

### Levofloxacin residue depletion and hematological impact in broiler chickens following repeated intramuscular administration

Over recent years, food production systems have shifted toward large-scale operations. One consequence of this industrialization has been the increased use of veterinary drugs to treat, control, or prevent infections in food-producing animals^19^. However, this practice raises concerns regarding drug residues in animal tissues and the associated risk of antimicrobial resistance and other adverse effects in humans consuming such products^20^. The present findings are consistent with previous reports highlighting the potential of probiotics as an effective tool in controlling enteric infections in poultry. In particular, *Enterococcus faecium* (M74) has been shown to protect broilers against experimentally induced necrotic enteritis, improving growth performance, enhancing intestinal health, and markedly reducing lesion severity^13^. Such probiotic effects, as reported by^21^ support the concept that targeted modulation of the gut microbiota can contribute to the reduction of pathogenic bacterial loads and improve overall flock health, complementing conventional therapeutic and preventive measures.

To safeguard public health, the World Health Organization (WHO) and the Food and Agriculture Organization (FAO) have established maximum residue limits (MRLs) for veterinary drugs, pesticides, and other chemicals in edible tissues of treated animals.

Fluoroquinolones, including levofloxacin, are commonly used in poultry for their broad-spectrum activity. The recommended withdrawal period for quinolones is approximately 4–5 days, ensuring residue levels fall below established MRLs^22–24^. According to the European Commission^25^, the MRLs for quinolones in broilers are 100 µg/kg for muscle, fat, and skin,200 µg/kg for liver, and 300 µg/kg for kidney, reflecting their pharmacokinetics and tissue distribution.

This study aimed to evaluate the residue depletion profile of levofloxacin in broiler chicken tissues—liver, breast muscle, and kidney—following intramuscular administration at 10 mg/kg body weight once daily for four consecutive days. High-performance liquid chromatography (HPLC) was employed to measure drug residues, ensuring that the tissues meet safety criteria for human consumption.

All groups were handled under similar conditions to minimize variability due to stress. However, oxidative stress markers were significantly altered only in the treated groups, indicating that the observed changes were more likely induced by levofloxacin rather than handling stress^26^.

This study did not extend beyond Day 9; therefore, long-term recovery could not be assessed. We recommend further investigations that include follow-up evaluations beyond this period to confirm complete physiological normalization.

The persistence of fluoroquinolone residues and their pharmacokinetic behavior have been widely studied in recent years. Rahman^18^ demonstrated the simultaneous detection of multiple antibiotics in chicken tissues using HPLC, confirming its reliability in food safety monitoring. Similarly, Anwar et al.^27^ emphasized the potential health effects of dietary antibiotics on poultry gut health, reinforcing the need for strict residue depletion studies such as the present work.

## Results and discussion

No observable clinical signs such as lethargy, diarrhea, reduced feed intake, or abnormal behavior were recorded during the study. The hematological and biochemical changes detected were subclinical and transient in nature.

Although the current study supports a 9-day withdrawal period under controlled laboratory conditions, real-world field settings may introduce variables such as stress, feed interactions, and disease that could influence drug depletion. Therefore, further validation under commercial farm conditions is recommended ^9^.

### Kidney tissue residues

**Fig. 15:**
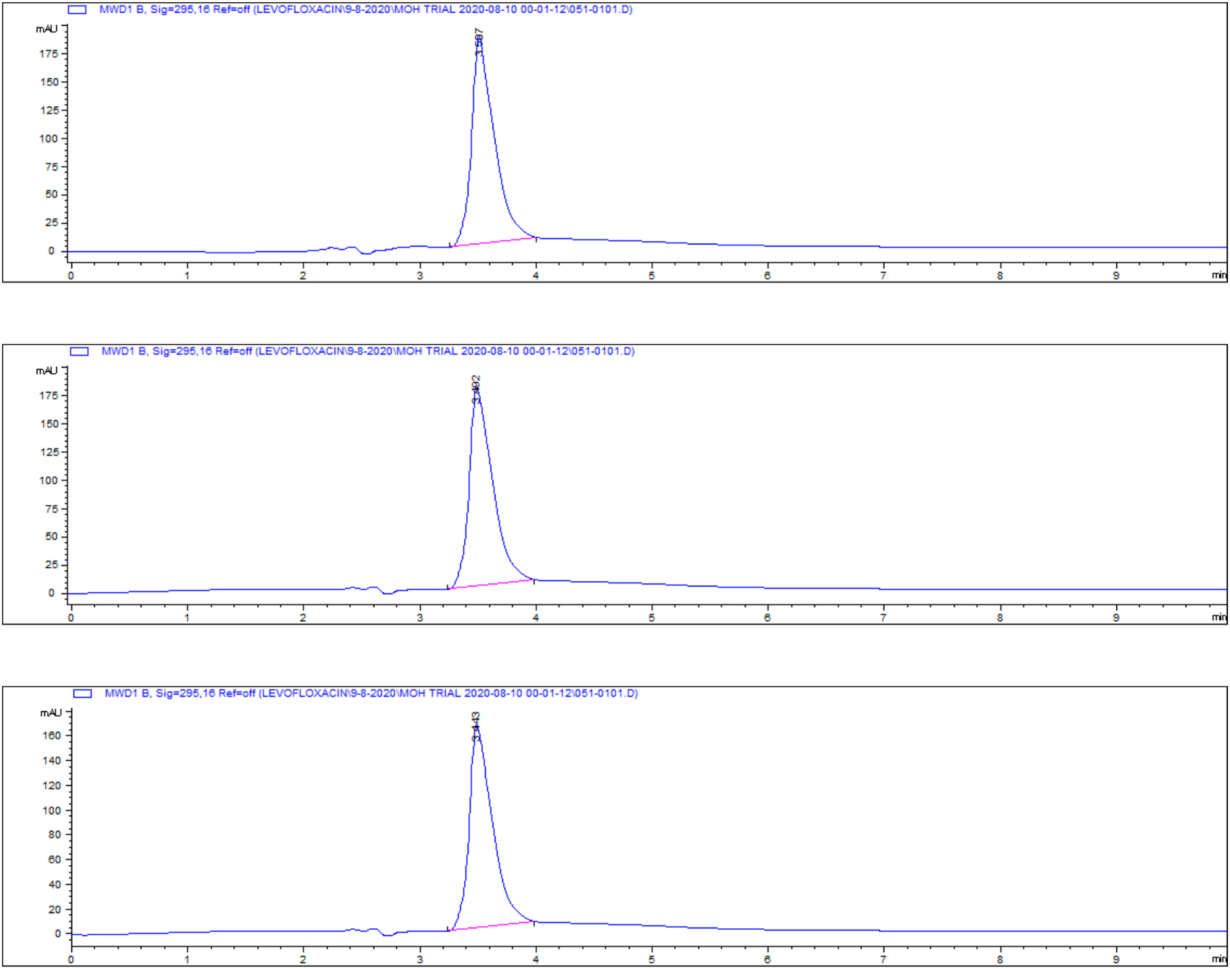
liquid chromatogram of levofloxacin extract of kidney 5th day post last day of administration after throughout the treatment course intramuscular dose (10 mg/kg.b.wt).

**Fig. 16:**
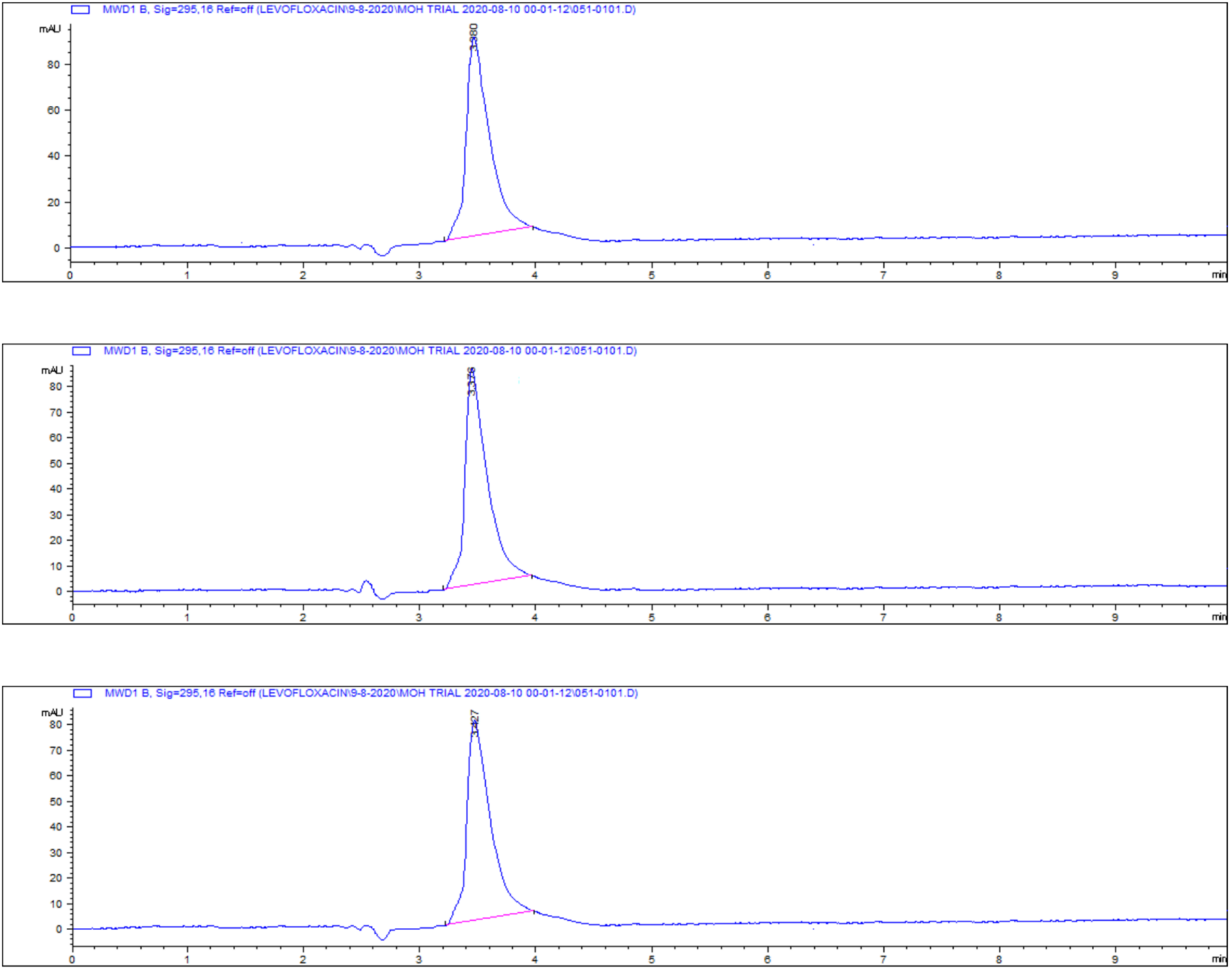
Liquid chromatogram of levofloxacin extract of kidney 7th day post last day of administration after throughout the treatment course intramuscular dose (10 mg/kg.b.wt).

**Fig. 17:**
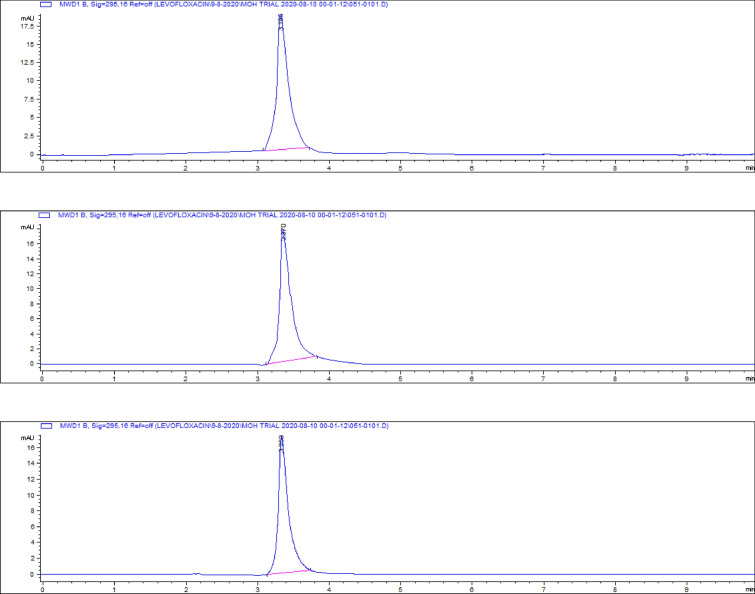
liquid chromatogram of levofloxacin extract of kidney 9th day post last day of administration after throughout the treatment course intramuscular dose (10 mg/kg.b.wt).

**Fig. 18:**
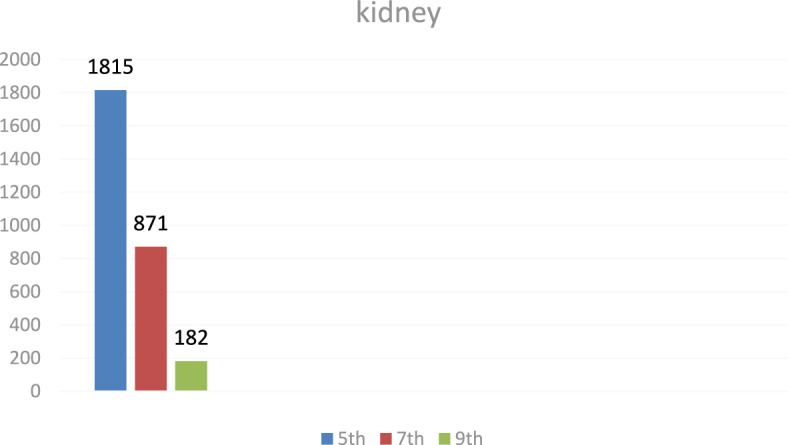
Residual withdrawal curve of levofloxacin in kidney post administration after throughout the treatment course intramuscular dose (10 mg/kg.b.wt).

The highest residue concentrations were detected in kidney tissues: 1815 ± 60.91 μg/kg on day 5, 871 ± 26.01 μg/kg on day 7, and 182 ± 4.35 μg/kg on day 9, as demonstrated by the representative chromatograms in Figs. 15, 16, 17. The depletion curve of levofloxacin residues in kidney tissues over the 9-day period is shown in Fig. 18, while the quantitative data are summarized in Table 2. These results corroborate previous research^28^^,^^29^ and underscore the kidney as a critical excretory organ where levofloxacin tends to accumulate. Similar pharmacokinetic observations regarding drug distribution in broiler tissues were also reported for lincomycin in cases of Clostridium perfringens infection^30^.

**Table 2:** Levofloxacin residues in poultry kidney post administration after 4 consecutive days intramuscular dose (10 mg/kg.b.wt).

Days Post administration	Residual Level (µg/kg)
5th	1815 ± 60.91
7th	871 ± 26.01
9th	182 ± 4.35

Values are mean ± SE (n = 10). (p < 0.05, one-way ANOVA followed by Tukey’s test). Units: µg/kg.

### Liver tissue residues

**Fig. 19:**
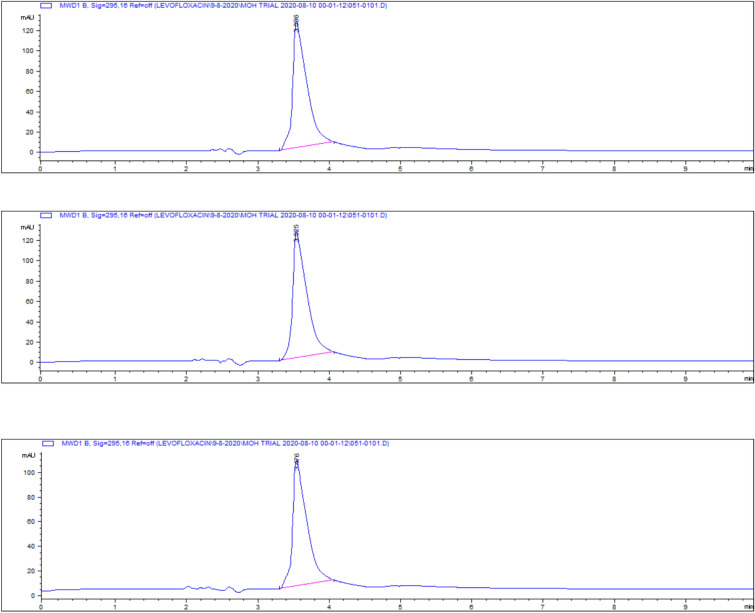
liquid chromatogram of levofloxacin extract of liver 5th day post last day of administration after throughout the treatment course intramuscular dose (10 mg/kg.b.wt).

**Fig. 20:**
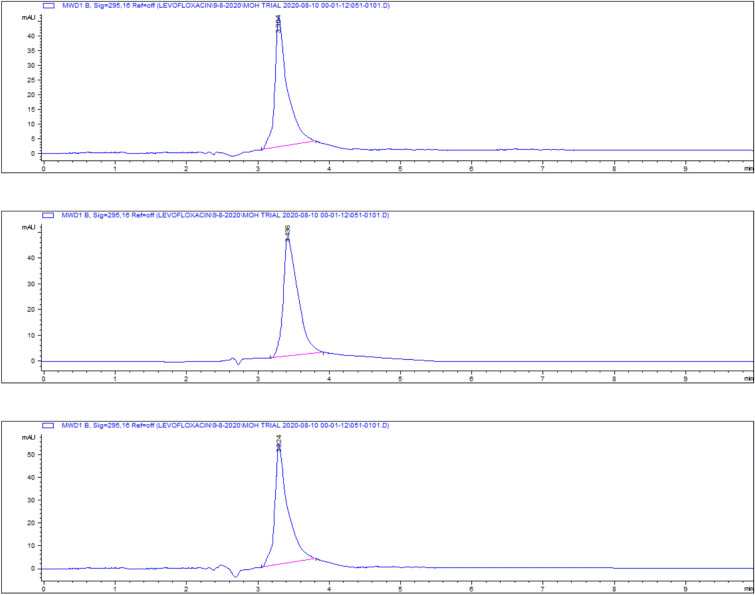
liquid chromatogram of levofloxacin extract of liver 7th day post last day of administration after throughout the treatment course intramuscular dose (10 mg/kg.b.wt).

**Fig. 21:**
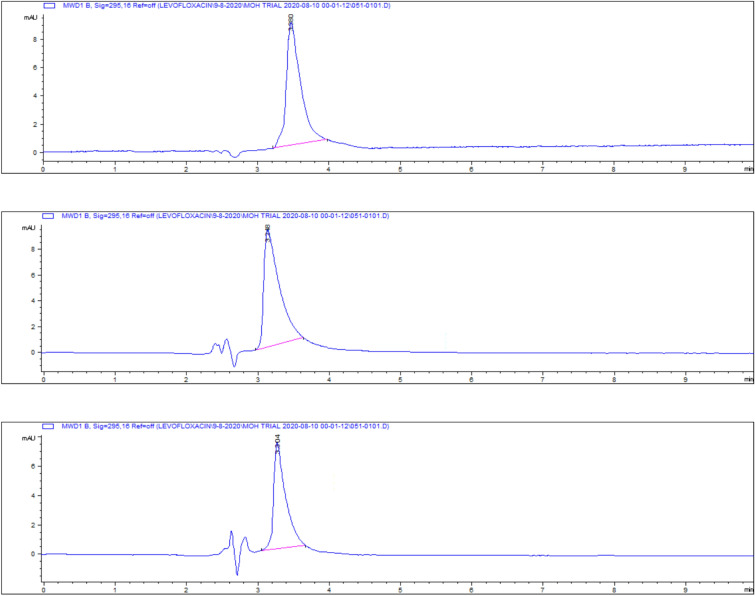
liquid chromatogram of levofloxacin extract of liver 9th day post last day of administration after throughout the treatment course intramuscular dose (10 mg/kg.b.wt).

**Fig. 22:**
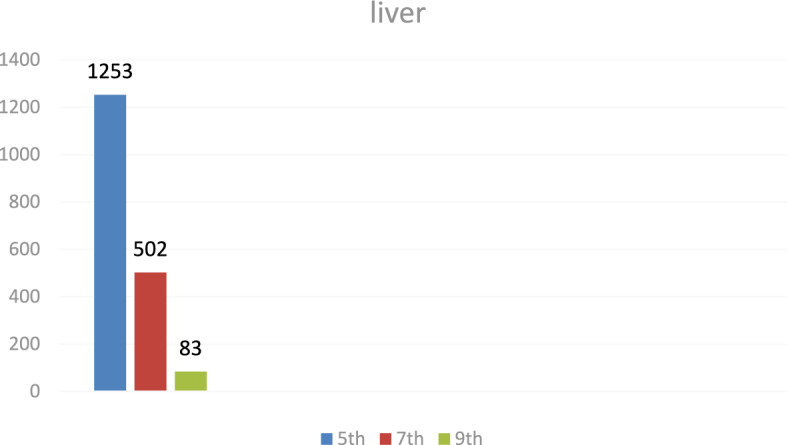
Residual withdrawal curve of levofloxacin in liver post administration after throughout the treatment course intramuscular dose (10 mg/kg.b.wt).

On day 5 post-treatment, the mean levofloxacin residue in liver samples was 1253 ± 70.48 μg/kg, decreasing to 502 ± 25.11 μg/kg by day 7 and 83 ± 4.61 μg/kg by day 9. These concentrations correspond to the chromatographic profiles presented in Figs. 19, 20, 21, while the levofloxacin depletion trend in liver tissue is illustrated in Fig. 22. The numerical data are listed in Table 3. These results are consistent with previous studies reporting high initial residues in liver tissue (e.g., 1196 μg/kg) that decline significantly by day 9 (24 μg/kg)^23^. Similar findings were observed by Kyuchukova et al.^31^^,^ Devada et al.^32^, and Anadon et al.^22^, affirming the liver’s role as a primary site of accumulation for quinolones.

**Table 3:** Levofloxacin residue in poultry Liver post administration after 4 consecutive days intramuscular dose (10 mg/kg.b.wt).

Days Post administration	Residual Level (µg/kg)
5th	1253 ± 70.48
7th	502 ± 25.11
9th	83 ± 4.61

Values are mean ± SE (n = 10). (p < 0.05, one-way ANOVA followed by Tukey’s test). Units: µg/kg.

### Breast muscle residues

**Fig. 23:**
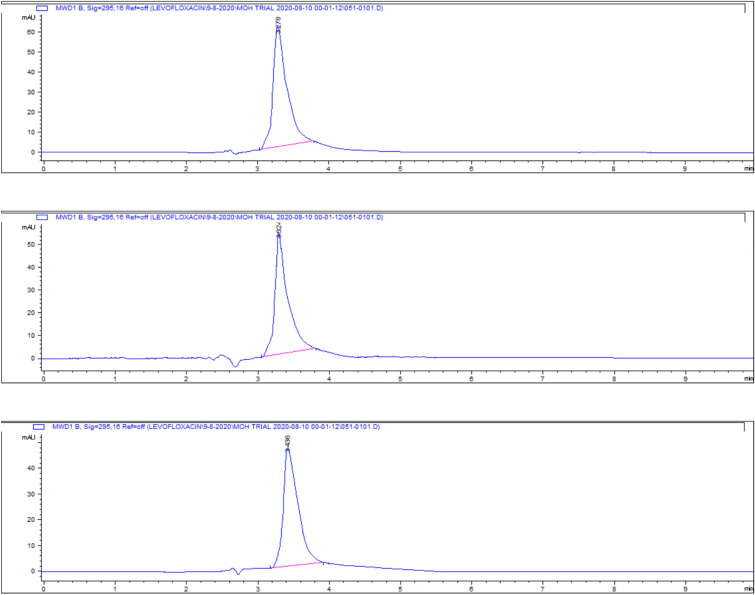
liquid chromatogram of levofloxacin extract of breast muscle5th day post last day of administration after throughout the treatment course intramuscular dose (10 mg/kg.b.wt).

**Fig. 24:**
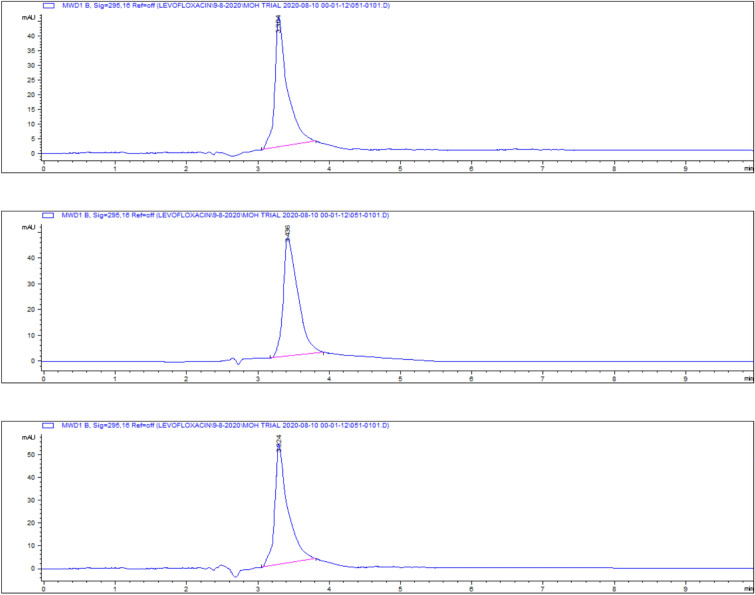
liquid chromatogram of levofloxacin extract of breast muscle 7th day post last day of administration after throughout the treatment course intramuscular dose (10 mg/kg.b.wt).

**Fig. 25:**
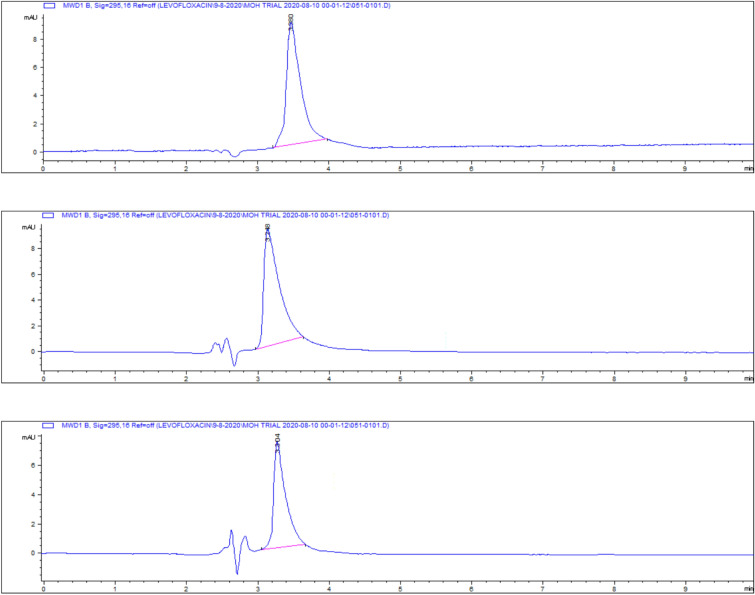
liquid chromatogram of levofloxacin extract of breast muscle 9th day post last day of administration after throughout the treatment course intramuscular dose (10 mg/kg.b.wt).

**Fig. 26:**
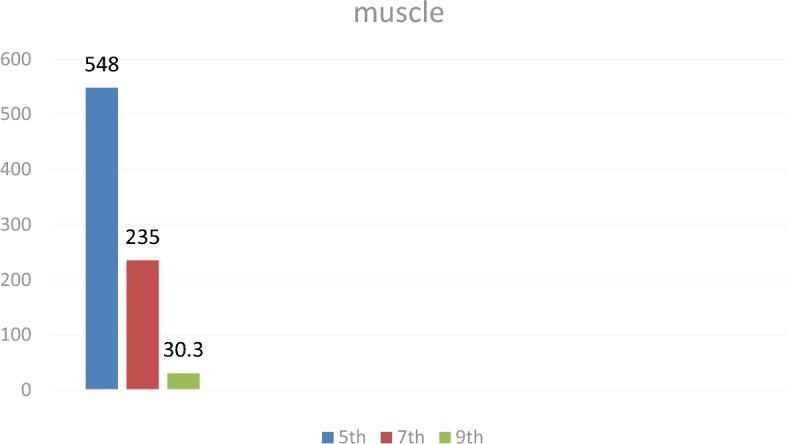
Residual withdrawal curve of levofloxacin in breast muscle post last day of administration after throughout the treatment course intramuscular dose (10 mg/kg.b.wt).

Levofloxacin residues in breast muscle were 548 ± 39.50 μg/kg on day 5, decreasing to 235 ± 17.45 μg/kg on day 7 and 30.3 ± 18.55 μg/kg on day 9. The corresponding chromatograms for muscle tissue across these days are shown in Figs. 23, 24, 25, and the depletion curve is illustrated in Fig. 26. Residue values are tabulated in Table 4. These results align with earlier findings^28^^,^^31^^,^^33^ that showed muscle tissues clear levofloxacin residues more quickly than liver or kidney tissues.

**Table 4:** Levofloxacin residue in poultry muscle post administration after 4 consecutive days intramuscular dose (10 mg/kg.b.wt).

Days Post administration	Residual Level (µg/kg)
5th	548 ± 39.50
7th	235 ± 17.45
9th	30.3 ± 18.55

Values are mean ± SE (n = 10). (p < 0.05, one-way ANOVA followed by Tukey’s test). Units: µg/kg.

### Final tissue comparison and safety

**Fig. 27:**
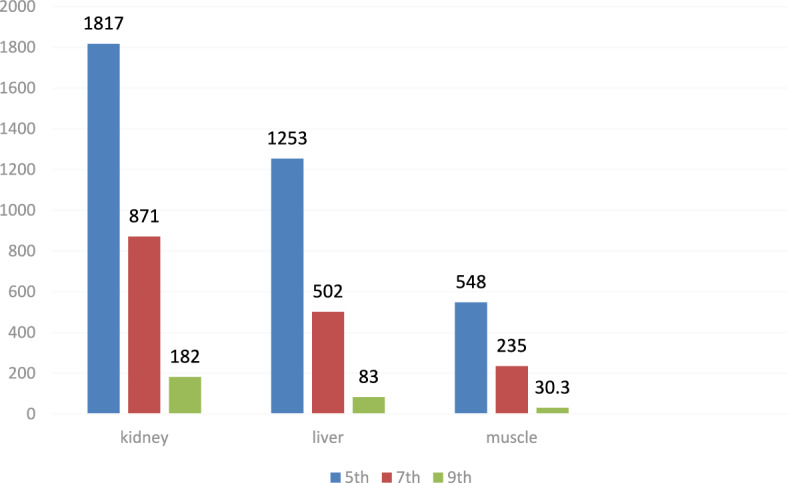
Residual withdrawal curve of levofloxacin in kidney, liver and muscle of poultry post administration after throughout the treatment course intramuscular dose (10 mg/kg.b.wt).

By day 9, residue levels in all tissues—muscle, liver, and kidney—had decreased below the European Commission’s MRLs, confirming that a withdrawal period of 9 days ensures food safety. A comparative summary of levofloxacin depletion across all tissues is presented in Fig. 27, with consolidated residue data and withdrawal timelines shown in Table 5.

**Table 5:** Levofloxacin residues level in poultry (kidney, liver, breast muscle) post administration after 4consecutive days intramuscular dose (10 mg/kg.b.wt).

Days post administration	Residual level (µg/kg)		
	Kidney	Liver	Muscle
5th	1815 ± 60.91	1253 ± 70.48	548 ± 39.50
7th	871 ± 26.01	502 ± 25.11	235 ± 17.45
9th	182 ± 4.35	83 ± 4.61	30.3 ± 18.55

Values are mean ± SE (n = 10). (p < 0.05, one-way ANOVA followed by Tukey’s test). Units: µg/kg.

### Hematological effects

1. ALT Elevation:

The significant elevation in ALT levels observed in treated broiler chickens suggests transient hepatic stress, potentially due to the hepatic metabolism of levofloxacin. ALT is a liver-specific enzyme, and its increase may reflect hepatocellular injury or drug-induced oxidative burden.

This observation is consistent with findings by Elkomy et al.^34^, who reported increased liver enzyme activities following administration of fluoroquinolones in poultry.

2. AST Elevation:

Increased AST levels further support the notion of hepatic strain. While AST is not liver-specific, its rise in conjunction with ALT suggests a systemic impact on liver function.

Similar elevations were noted in the study by Abou Elazab et al.^35^, where enrofloxacin administration altered hepatic biomarkers in broilers.

3. Urea and Creatinine Changes:

The observed changes in urea and creatinine levels may indicate a temporary disturbance in renal function, possibly due to drug clearance pathways or oxidative stress-induced nephrotoxicity.

These findings align with research by Goudah and Hasabelnaby^36^, who reported renal parameter fluctuations after fluoroquinolone treatment in avian species.

4. Hematological Changes (e.g., RBCs, Hb, PCV):

In addition to residue analysis, hematological parameters were assessed. Results revealed:

- A significant (p < 0.05) decrease in total erythrocytic count at days 5 and 7 post-treatment, with a non-significant decrease on day 9.
- A significant increase in total leukocytic count at days 5 and 7, with a non-significant increase on day 9.
- A significant reduction in hemoglobin concentration at days 5 and 7, followed by a non-significant increase at day 9.

These findings suggest a transient hematological disturbance following levofloxacin administration, possibly due to its inhibitory effects on hematopoietic cells or bone marrow function^37^^,^^38^. Similar effects have been reported for other quinolones, including danofloxacin and enrofloxacin, which caused reductions in hemoglobin content, erythrocyte counts, and packed cell volume while inducing leukocytosis^39^^,^^40^^,^^41^.

Reductions in RBC count, hemoglobin concentration, and PCV may reflect bone marrow suppression or hemolytic effects associated with fluoroquinolone exposure."

"El-Shazly et al.^42^ similarly documented hematological alterations in chickens treated with enrofloxacin, suggesting a class effect of fluoroquinolones on erythropoiesis.

5. Oxidative Stress Markers:

Alterations in oxidative stress markers such as MDA and total antioxidant capacity (TAC) indicate that levofloxacin may generate reactive oxygen species, contributing to oxidative tissue damage."

This is in accordance with the work of Zaki et al. (2018), who demonstrated oxidative imbalance in broilers following fluoroquinolone exposure.

6. Reversibility of Changes by Day 9:

The return of biochemical and hematological markers to near-normal values by day 9 post-treatment suggests that the physiological disruptions were reversible. This supports the safety of a 9-day withdrawal period under controlled conditions.

However, field variability may necessitate further investigation under commercial settings to validate this interval.

This study not only provides withdrawal guidelines for levofloxacin in broilers but also highlights the transient hematological and biochemical changes that should be monitored during therapeutic use.

## Conclusion

The current study indicates that levofloxacin residue level in the 5thday post last day treatment were kidneys 1815 ± 60.91 ug/kg, liver 1253 ± 70.48 ug/kg then breast muscle 548 ± 39.50 ug/kg. In the 7th day post last day treatment, the residues minimized in all organs and become 871 ± 26.01ug/kg, 502 ± 25.11 ug/kg and 235 ± 17.45 ug/kg in kidneys, liver and breast muscle, respectively. Moreover, in the 9th day post treatment, Levofloxacin residues were reduced to 182 ± 4.35from in kidneys, 83 ± 4.61 for liver and 30.3 ± 18.55 ug/kg in breast muscle post administration after throughout the treatment course intramuscular dose (10 mg/kg.b.wt). and according to The European Union has defined MRL for quinolones. In broilers, the defined MRL is 100 µg/kg for muscle, fat, and skin; 200 µg/kg for the liver; and 300 µg/kg for the kidneys.

Levofloxacin (levoxin) used intramuscular administration once daily throughout the treatment course at a dosage of 10 mg/kg b.wt.

Thus, under the controlled conditions of this experiment, levofloxacin residues in kidney, liver and breast muscle declined below the established EU MRLs by Day 9 post-treatment, suggesting a 9-day withdrawal period may be adequate. However, variability in field conditions (disease status, feed, stress, dosage formulations) could alter depletion; therefore, further validation under commercial farm conditions is recommended.

## Supplementary Information

Supplementary Information 1.

Supplementary Information 2.

Supplementary Information 3.

## Data Availability

The datasets generated/analyzed during the current study are available from the corresponding author on reasonable request.
