# Supplementary material for: Pharmacological evaluation of levofloxacin residue depletion and hemato-biochemical alterations in broiler chickens using a validated HPLC method
Source: Sci Rep. 2025 Sep 12;15:32496. doi: 10.1038/s41598-025-17575-0 (PMC12432151; doi:10.1038/s41598-025-17575-0)
Supplement: Supplementary file 1 — Supplementary Information 1. [file 41598_2025_17575_MOESM1_ESM.doc]

**SUMMARY**

**The purpose of this study is to**

- Determine the withdrawal time of levofloxacin from liver, kidneys and breast muscles of broilers after intra­muscular injection (I/M) of the drug (10 mg/kg for 4 consecutive days) by using HPLC .
- Evaluate some hematological parameters (CBC) after intramuscular injection (I/M) of the drug (10 mg/kg for 4 consecutive days).
- Evaluate some biochemical parameters as liver function enzymes (AST , ALT), kidney function enzymes (blood serum urea, creatinine) and antioxidants enzymes (SOD, GPX , CAT ) in blood serum .

**Experimental design:**

The study was conducted in twenty days (n= 40) healthy Rock-3 (IR-3), a strain of male broiler chicken in 4 groups equal 10 at each at a weight 1 kgm ±50 gm weight developed in Elabrar poultry company, Abohamad, Egypt.

The chickens were classified in 4 groups :

**1^st^ group** : Conrol chicks non treated.

**2^nd^ group**  : Injected intramuscularly by levofloxacin ( levoxin vial) at a dose of (10 mg/kg.b.wt) for 4 consecutive days then slaughtered on the 5^th^ day post injection .

**3^rd^ group** : Injected intramuscularly by levofloxacin ( levoxin vial) at a dose of (10 mg/kg.b.wt) for 4 consecutive days then slaughtered on the 7^th^ day post injection .

**4^th^ group** : Injected intramuscularly by levofloxacin ( levoxin vial) at a dose of (10 mg/kg) for 4 consecutive days then slaughtered on the 9^th^ day post injection.

**Sampling**:

- **Samples collected for detection of levofloxacin residues :**

100 gram of breast muscle, 50 gram of liver and 5 gram of kidney samples obtained from every chicken from all groups.

- **Samples for hematological analysis and blood serum for biochemical analysis :**

1. Whole blood for blood analysis
2. Serum for biochemical analysis

**1- Hematological parameters:-**

Healthy chickens administered levofloxacin for 4 consecutive days displayed significant decrease in erythrocytic count, hemoglobin content beside significant leukocytosis on 5^th^ and 7^th^ day post last day of injection accompanied with insignificant effect on blood picture on 9^th^ day post injection when compared with control chickens.

1. **Serum biochemical parameters:**

**A) Liver enzymes:**

The obtained results in this study revealed that apparently healthy chickens received levofloxacin for 4 consecutive days produced a significant increase in serum AST, ALT and ALP on 5^th^ and 7^th^ day post last day of treatment associated with insignificant increase on 9^th^ days post treatment when compared with control chickens.

**B) Kidney function:**

Apparently healthy chickens received levofloxacin for 4 consecutive days revealed an increase in the level of urea and creatinine on 5^th^ and 7^th^ day post injection and non significant increase on 9^th^ day post injection when compared with control chickens.

**C)Estimation of (Enzymatic Antioxidants )**

Apparently healthy chickens received levofloxacin for 4 consecutive days revealed an increase in the level of SOD, GPX and CAT on 5^th^ and 7^th^ day post injection and non significant increase on 9^th^ day post injection when compared with control chickens.

1. **Levofloxacin residues:**

The current study indicates that levofloxacin residue level on the 5^th^ day post last day treatment was in kidneys 1815± 60.91 ug/kg, liver 1253± 70.48 ug/kg then breast muscle 548 ± 39.50 ug/kg. On the 7^th^ day post last day treatment, the residues minimized in all organs and become 871± 26.01ug/kg, 502 ± 25.11 ug/kg and 235 ± 17.45 ug/kg in kidneys, liver and breast muscle, respectively. Moreover, on the 9^th^ day post treatment, the residues minimized to 182±4.35 in kidneys, 83±4.61 in liver and 30.3 ± 18.55 ug/kg in breast muscle post administration after 4 consecutive days intramuscular dose (10 mg/kg.b.wt). According to the European Union has defined MRL for quinolones in broilers, the defined MRL is 100 µg/kg for muscle, fat and skin ; 200 µg/kg for the liver and 300 µg/kg for the kidneys.

Levofloxacin (levoxin) intramuscularly administrated once daily for 4 consecutive days at a dosage (10 mg/kg b.wt.).

So, Breast muscle, liver and kidneys of chicken considered safe for human consumption from the 9^th^ day post last day of treatment .

**CONCLUSION**

***Conclusion***

⮊ The Breast muscle, liver and kidneys of chicken considered safe for human consumption from 9^th^ day post last day of treatment .

⮊ So withdrawal peroid of levofloxacin after 4-5 day post treatment .
