# Supplementary material for: Pharmacological evaluation of levofloxacin residue depletion and hemato-biochemical alterations in broiler chickens using a validated HPLC method
Source: Sci Rep. 2025 Sep 12;15:32496. doi: 10.1038/s41598-025-17575-0 (PMC12432151; doi:10.1038/s41598-025-17575-0)
Supplement: Supplementary file 3 — Supplementary Information 3. [file 41598_2025_17575_MOESM3_ESM.pdf]

Supplementary Material – HPLC Analytical Method

This supplementary document provides detailed information about the high-performance liquid chromatography (HPLC) method used for the analysis of levofloxacin residues in broiler chicken tissues.

---

1. HPLC System and Apparatus

- Instrument: Shimadzu LC-20 liquid chromatograph (Kyoto, Japan)
- Detector: UV detector (SPD-20A)
- Injection system: Rheodyne injection valve with a 20 µL sample loop

2. Chromatographic Conditions

- Column: C18 reversed-phase column (250 mm × 4.6 mm, 5 µm particle size; Thermo Fisher Scientific, USA)
- Mobile phase: 0.05 M phosphate buffer (pH 3.0) and acetonitrile in a ratio of 80:20 (v/v)
- Flow rate: 1 mL/min
- Detection wavelength: 295 nm
- Total runtime: 10 minutes per sample
- Column temperature: Room temperature

3. Solvent Preparation and Filtration

- All solvents were HPLC-grade.
- The mobile phase was prepared freshly, mixed thoroughly, and degassed using an ultrasonic bath for 15 minutes.
- Prior to use, all solvents and samples were filtered through 0.22 µm syringe filters (Millipore, USA).

4. Sample Preparation

- Tissue samples were homogenized using a mechanical homogenizer.
- A suitable volume of acetonitrile was added to the tissue sample and vortexed.
- The mixture was centrifuged, and the supernatant was collected and filtered before injection.

5. Chemicals and Reagents

- Levofloxacin standard was obtained from a certified pharmaceutical supplier.
- Acetonitrile and phosphate buffer components were purchased from Sigma-Aldrich (USA).

6. Method Validation Summary

- **Linearity**: The calibration curve showed linearity over the concentration range used, with $r^2 > 0.999$.
- **Limit of Detection (LOD)**: 0.005 µg/g
- **Limit of Quantification (LOQ)**: 0.01 µg/g
- **Recovery**: 87–93% across different tissues
- **Repeatability**: Relative standard deviation (RSD) < 5% for intra-day and inter-day precision

Section 1: HPLC Equipment and Reagents

1- **High Performance Liquid Chromatography: - (HPLC)**

1) Constant liquid chromatography Quat pump G1311A Serial No.DE60556457 , Agilent company, USA.

2) An autosampler plus G1329A Serial No. DE60556754 , Agilent company, USA.

3) Variable Flouroscence detector Serial No. DE60555416, surveyor, Agilent company, USA.

4) Multi Wave Detector Serial No. DE60555274 , Agilent company, USA.

5) A software chromo quest 5, surveyor, Agilent company, USA

for data analysis automatically.

2-Analytical column:-

The chromatographic column was a reversed-phase column (C18 column

(4.6 mm i.d, 250mm, 5 µm), Serial No. USAU044986 ,Agilent company, USA.

3-Electronic digital balance: - (Vibra, Shinko Denshi Co., LTD, Japan)

4-PH meter: - (Jenway)

5-Moulinette mincer: - (Kika® werke, Germany)**.**

6- Automatic pipettes: - (Eppendorf Research) (10 – 100 µl) and (100 – 1000 µl).

7- Vortex mixer: - (Bibby sterelin ltd, Stone, UK).

8-Glass wares: (grade A, Germany)

Volumetric flasks (10, 25, 50, 100, 500, 1000µl), cylinders (100, 250 ml),

glass pipettes (5-10 ml) and HPLC vials.

9-Plastic insulin syringes (1 ml).

10-Stoppered poly propylene centrifuge tubes (15, 50 ml).

11-Orbital shaker.

12- SPE vacuum manifold.

13- Solid-phase extraction (SPE) columns: Bond Elut C18 (500 mg, 3 or 6 mL; Varian, Les Ulis, France).

14- High speed cooling centrifuge: - (Centurion, USA).

15-Acrodiscs (syringe filters): Millex HV13 filters (0.45 μm), 13 mm id

(Millipore, Saint Quentin Yvelines, France).

Chemical reagents:

1-Organic solvents :(HPLC grade)

Methanol, trifluoroacetic acid and potassium hydroxide obtained from Fisher Scientific Co, Fairlawn, NJ, UK. Formic acid and Glacial acetic acid purchased from Merk Co, Darmstadt, Germany. Acetonitrile purchased from J.T. Baker Co, Deventer, Netherlands. Triethylamine and phosphoric acid were purchased from Sigma Aldrich Co, Germany. perchloric acid obtained from s d fine-chem Limited, Mumbai, India. Sodium hydroxide purchased from Honeywell Co, Germany.

2- **Water:** HPLC grade.
3-Chemicals:

Granular potassium dihydrogen phosphate monobasic (KH2PO4) obtained from Sham Lab, Syria. Ammonium acetate obtained from Fisher Scientific Co, Fairlawn, NJ, UK. Dithioerythritol, iodoacetamide, potassium chloride and sodium chloride are from Sigma Aldrich Co, UK. Calcium chloride purchased from Merk Co, Darmstadt, Germany. Sodium borate obtained from Loba chemie PVT, Mumbai, India.

Section 2: General Laboratory Instruments

- Centrifuge: (MLWT 52-1-Germany) used for separating humour.
- Deep freezer (-20oC) for keeping humour samples (Deepfreezer (Ideal CoARE)
- Electrical balance: (Sartorius GOH Ingen, sort 1401 A, W Germany) with a soap wt. 3600-0 wt and min wt. 0.1gm.
- Electrical water bathtub (fisher tissue TN flotation bathtub model dl34) was used for determination of organic chemistry values.
- Hot air oven: (Memmert, w Germany) was used for sterilization of equipments
- photometer spectronic 20D was used for quantitative analysis determination of organic chemistry values (Spectrophotometer spectronic 20D (Milton Roy Comp).
- different experimental equipments: Beakers, bottles, cotton, cowl slide, glass slides, flasks, forceps, gauze, graduate, knifes, petri dishes, pipettes, scissors, stop watches, syringes.
